# Effects of Intragastric *Helicobacter pylori* Distribution on Clinical Presentation, Upper Gastrointestinal Endoscopy, Esophageal Manometry, and pH–Impedance Metrics

**DOI:** 10.3390/jcm14196818

**Published:** 2025-09-26

**Authors:** Ayça Eroğlu Haktanır, Altay Çelebi

**Affiliations:** Department of Gastroenterology, Faculty of Medicine, Kocaeli University, Kocaeli 41001, Turkey; altaycelebi@yahoo.com

**Keywords:** *Helicobacter pylori*, gastroesophageal reflux disease, GERD, intragastric distribution, esophageal motility, impedance–pH monitoring, mean nocturnal baseline impedance, DeMeester score, acid exposure time, AET, chronic laryngitis, nausea

## Abstract

**Background:** The relationship between *Helicobacter pylori* (*H. pylori*) infection and gastroesophageal reflux disease (GERD) remains a topic of ongoing debate. In particular, the intragastric distribution of *H. pylori*—whether localized in the antrum or corpus—may influence gastric acid secretion and esophageal physiology in different ways. However, its potential effects on esophageal motility and reflux parameters have not been comprehensively evaluated using combined diagnostic tools. This study aimed to assess whether *H. pylori* positivity, based on its histologically confirmed intragastric localization, is associated with alterations in endoscopic, manometric, and reflux monitoring findings in patients with typical GERD symptoms. **Methods:** This retrospective study included 213 patients with typical reflux symptoms who underwent upper gastrointestinal endoscopy with gastric biopsies, high-resolution esophageal manometry (HREM), and 24 h multichannel intraluminal impedance–pH (MII-pH) monitoring. Based on histopathology, patients were classified into three groups: *H. pylori*-negative, antrum-predominant infection, and corpus-predominant infection. Clinical symptoms, endoscopic findings, reflux characteristics, and esophageal motility parameters were compared. **Results:** Of 213 patients, 90 were *H. pylori*-positive (60 antrum-predominant, 30 corpus-predominant). There were no significant differences between groups in terms of typical GERD symptoms, endoscopic esophagitis, DeMeester scores, acid exposure time, or mean nocturnal baseline impedance (MNBI). Nausea and chronic laryngitis were significantly more frequent in antral *H. pylori*-positive patients. Notably, contraction front velocity (CFV) was significantly lower in patients with antral *H. pylori* compared with *H. pylori*-negative individuals (*p* = 0.002), indicating subtle slowing of esophageal peristalsis. Although this reduction in CFV did not correlate with symptom severity or bolus clearance, it may represent early functional impairment of esophageal motility. **Conclusions:** Although *H. pylori* infection—particularly when antrum-predominant—is not associated with increased reflux burden or esophagitis, it may contribute to extra-esophageal symptoms and minor motility alterations such as reduced CFV. These findings suggest that routine *H. pylori* eradication in GERD patients may not be necessary solely based on reflux parameters. However, treatment decisions should be individualized based on symptom profiles and endoscopic findings, including the presence of peptic ulcers, premalignant gastric lesions, or a family history of gastric malignancy, in accordance with general *H. pylori* eradication criteria.

## 1. Introduction

*Helicobacter pylori* (*H. pylori*) is a Gram-negative, spiral-shaped bacterium and one of the most prevalent chronic infections worldwide. Its prevalence depends on factors such as age, geographic region, ethnicity, and socioeconomic status [1]. Global epidemiological data show a decline in *H. pylori* infection rates over the past four decades, from 58.2% to 43.1% [2]. However, the TURHEP study in Turkey reported a much higher prevalence of 82.5% [3].

*H. pylori* is well established as a causative agent of chronic gastritis, peptic ulcer disease, and gastric cancer. However, its role in gastroesophageal reflux disease (GERD) remains controversial. The intragastric distribution of *H. pylori*—whether predominantly in the antrum, corpus, or diffusely—can influence gastric acid secretion differently, which may in turn affect GERD pathogenesis. While some studies suggest that *H. pylori* may have a protective role against GERD by modulating acid output [4,5,6,7], others report symptom relief or even exacerbation following eradication therapy [8,9,10]. These conflicting findings reflect a complex, multifactorial interaction that remains poorly understood. Clarifying the impact of *H. pylori* localization on GERD pathogenesis is clinically relevant, as it may influence decisions regarding eradication therapy, particularly in patients presenting with overlapping reflux symptoms and documented infection. Despite numerous investigations, a unified conclusion has yet to be reached, underscoring the need for further research to clarify this intricate relationship.

Despite the extensive body of research on *H. pylori* and GERD, few studies have employed objective and comprehensive diagnostic tools—such as MII-pH and high-resolution esophageal manometry (HREM)—to explore this relationship [11,12]. Moreover, data on how the specific localization of *H. pylori* within the stomach affects reflux parameters remain scarce.

In this context, the present study aims to investigate the relationship between *H. pylori* infection and GERD, with a specific focus on the intragastric distribution of the bacterium (antral, corporal, or diffuse).

To best of our knowledge, this is the first study to assess the impact of *H. pylori* localization on reflux parameters using both MII-pH and HREM in the same patient cohort. Given the high national prevalence of *H. pylori* in Turkey, our results should be interpreted with caution when extrapolating to populations with lower infection rates. Regional differences in bacterial virulence factors (e.g., CagA/VacA status), host genetic susceptibility, and environmental influences may limit the generalizability of these findings [1,2,3,13,14]. Therefore, by correlating *H. pylori* distribution with demographic features, clinical symptoms, endoscopic findings, and functional test results, we aim to clarify its potential role in GERD pathogenesis and progression.

## 2. Materials and Methods

### 2.1. Patient Population

This retrospective study included patients who presented to the Department of Gastroenterology at Kocaeli University Faculty of Medicine between January 2019 and January 2021 with typical gastroesophageal reflux symptoms lasting at least six months. This study was conducted in accordance with the Declaration of Helsinki and Good Clinical Practice guidelines, ethical approval was obtained from the institutional review board, i.e., the Clinical Research Ethics Committee of Kocaeli University Faculty of Medicine (protocol no. 2024/369; approval date: 12 September 2024). Patient records were reviewed retrospectively. The inclusion criteria required patients to have a fully completed standardized reflux symptom questionnaire [15] documented in their files, to have undergone upper gastrointestinal (GI) endoscopy with biopsy samples taken from both the antrum and corpus, and to have complete and accessible data from HREM as well as MII-pH monitoring. In the GERD Q questionnaire, patients were asked about their history of *H.pylori* eradication therapy within the past year, and individuals who had received eradication treatment within the last three months were excluded from the study to minimize potential confounding effects related to recent therapy.

### 2.2. Patient Selection and Data Collection

A total of 238 patients presenting with symptoms suggestive of GERD were initially assessed for study eligibility. Of these, 115 patients were identified as *H. pylori* positive based on histopathological examination of gastric biopsy specimens obtained from the antrum and corpus during upper GI endoscopy. However, 25 patients from this group were excluded due to the following reasons: achalasia detected on esophageal manometry (*n* = 4), esophagogastric junction outflow obstruction (*n* = 2), a history of active malignancy (*n* = 2), MII-pH duration of less than 22 h (*n* = 4), the presence of severe systemic illness such as chronic kidney disease, congestive heart failure, or liver cirrhosis (*n* = 5), a history of upper GI surgery (*n* = 5) and hiatal hernia larger than 3 cm (*n* = 3). After these exclusions, 90 *H. pylori*-positive and 123 *H. pylori*-negative patients were included in the final analysis. Demographic data including age, sex, body mass index (BMI), smoking and alcohol use, medical history, and regular medications were extracted from patient files and reflux symptom questionnaires [15]. These forms also documented the frequency, severity, and duration of reflux-related symptoms. Typical reflux symptoms included heartburn, regurgitation, and non-cardiac chest pain. Atypical symptoms were defined as belching, nausea, vomiting, hiccups, chronic cough, dyspnea, hoarseness, and dyspeptic complaints. All typical GERD symptoms, such as heartburn and regurgitation, were systematically assessed and recorded for frequency and severity using a standardized reflux questionnaire [15]. Symptom frequency was graded based on patient-reported data as follows: no symptoms or less than once per week (1 point), once per week (2 points), two to three times per week (3 points), and four or more times per week (4 points). Symptom severity was scored as follows: none (0), mild (not noticeable unless attention is drawn to it; 1 point), moderate (bothersome but does not interfere with daily life; 2 points), severe (interferes with daily life; 3 points), and very severe (significantly impairs daily life; 4 points). MII-pH results were evaluated according to the Lyon Consensus 2.0 [16], esophagitis severity using the Los Angeles (LA) classification [17], and HREM findings based on the Chicago Classification v4.0 [18]. A definitive GERD diagnosis was made in patients with an acid exposure time (AET) ≥ 6% and LA grade B–D esophagitis, or in the presence of Barrett’s esophagus, peptic stricture, or abnormal pH–metry findings accompanied by troublesome symptoms. Patients who did not meet these definitive criteria but had LA grade A esophagitis, AET between 4 and 6%, 40–80 reflux episodes (REs), or MNBI between 1500 and 2500 ohms, and no characteristic manometric abnormalities, were classified as borderline. Supportive findings for GERD included hiatal hernia, positive symptom–reflux correlation, total REs > 80, hypotensive esophagogastric junction, ineffective esophageal motility (IEM), absent peristalsis, or MNBI < 1500 ohms [16]. Prior to the administration of MII-pH and HREM, proton pump inhibitors (PPIs) were discontinued for a minimum of 2 to 4 weeks. Additionally, medications known to affect lower esophageal sphincter (LES) pressure or gastrointestinal motility, such as nitrates, calcium channel blockers, prokinetics including metoclopramide, domperidone, and cisapride, as well as anticholinergics, were withheld for at least 48 to 72 h in accordance with established clinical protocols to minimize potential confounding effects on the test results.

### 2.3. Upper Gastrointestinal Endoscopy

In this retrospective study, patient records were reviewed for individuals who had previously undergone upper GI endoscopy due to upper GI symptoms, during which endoscopic biopsy samples were collected. Endoscopic findings consistent with reflux esophagitis were staged according to the LA classification. To diagnose *H. pylori* infection and assess its distribution within the gastric mucosa, five biopsy samples were collected during endoscopy, following the Sydney protocol: two from the antrum, one from the incisura angularis, and one each from the lesser and greater curvature of the corpus [19]. All biopsy specimens, approximately 3 × 3 mm in size, were obtained single-use biopsy forceps (Endo-Flex^®^, GmbH, Voerde, Germany), and endoscopic procedures were performed using the FUJIFILM EG-760R gastroscope (FUJIFILM Corporation, Tokyo, Japan).

### 2.4. Histopathological Evaluation and Detection of Helicobacter pylori

Gastric biopsy specimens obtained during endoscopy were fixed in 10% formalin, embedded in routine paraffin blocks, and stained with hematoxylin and eosin. The modified Giemsa staining method was used for the detection of *H. pylori*. Histopathological analysis of the antral and corpus biopsy samples was performed in accordance with the modified Sydney classification to assess the presence and severity of gastritis [20].

Mononuclear cell infiltration in the lamina propria, neutrophil activity, glandular atrophy, and *H. pylori* density were each graded on a scale from 0 (absent) to 3 (severe). All histopathological evaluations were performed by a single experienced pathologist, who was, blinded to clinical and endoscopic findings. Although *H. pylori* density was semi-quantitatively scored (0–3) based on the modified Sydney classification, statistical analysis was limited to a binary classification (positive/negative) due to the small number of patients in each density subgroup.

### 2.5. Esophageal pH Monitoring

All patients underwent 24-h MII monitoring using a multichannel, single-use Digitrapper^®^ VersaFlex-Z Disposable Impedance Catheter (Medtronic, Minneapolis, MN, USA). After nasal insertion of the catheter, the distal pH sensor was positioned and secured 5 cm above the LES to ensure accurate measurement. The pH catheter used for esophageal monitoring was calibrated prior to the procedure using buffer solutions at pH 1.0 and pH 7.0. Following calibration, the catheter was advanced transnasally with reference to the Z-line previously identified during upper GI endoscopy. Using a pH-guided placement technique, the catheter tip was positioned approximately 5 cm proximal to the Z-line. After confirming that the distal end of the catheter accurately detected intragastric pH, it was retracted and placed at the target point within the esophagus. Final positioning was confirmed under fluoroscopy. Recordings were maintained for at least 22 h and up to 24 h. To ensure consistency, all MNBI measurements were performed manually by a single experienced physician (Haktanir A. E). The MNBI was calculated by averaging impedance values from the distal esophageal channel (typically channel Z5, located 5 cm above the lower esophageal sphincter) during three separate 10-min intervals at approximately 1:00 a.m., 2:00 a.m., and 3:00 a.m., specifically during periods without swallowing or REs. In addition to standard impedance–pH parameters, symptom association probability (SAP) and symptom index (SI) were calculated where patient-reported symptom entries were available and met analysis criteria. SAP was considered positive if ≥95%, and SI was deemed positive if ≥50%, in accordance with established guidelines [16]. These indices were used to evaluate the temporal association between reflux episodes and patient-reported symptoms.

### 2.6. Esophageal Manometry Evaluation

Esophageal manometry tests were performed to assess esophageal motility following a minimum of 8 h of fasting. Measurements were obtained using a multichannel water-perfused manometry system integrated with Medical Measurement Systems (MMS) database software (version 9.3r; MMS B.V., Enschede, Overijssel, The Netherlands). The first five orifices of the catheter were spaced at 1 cm intervals, while the remaining channels were spaced 2 cm apart. The catheter was calibrated within a pressure range of 0–50 mmHg prior to insertion, then introduced transnasally and positioned to extend beyond the LES. Esophageal motor functions were interpreted according to the Chicago Classification v4.0 [18]. The analysis included intraluminal pressure changes during swallowing, amplitude, duration, and velocity of peristaltic waves in the esophageal body, and LES resting pressure. Based on these measurements, major and minor motility disorders were classified accordingly.

### 2.7. Esophageal Motility Assessment

**Figure 1:**
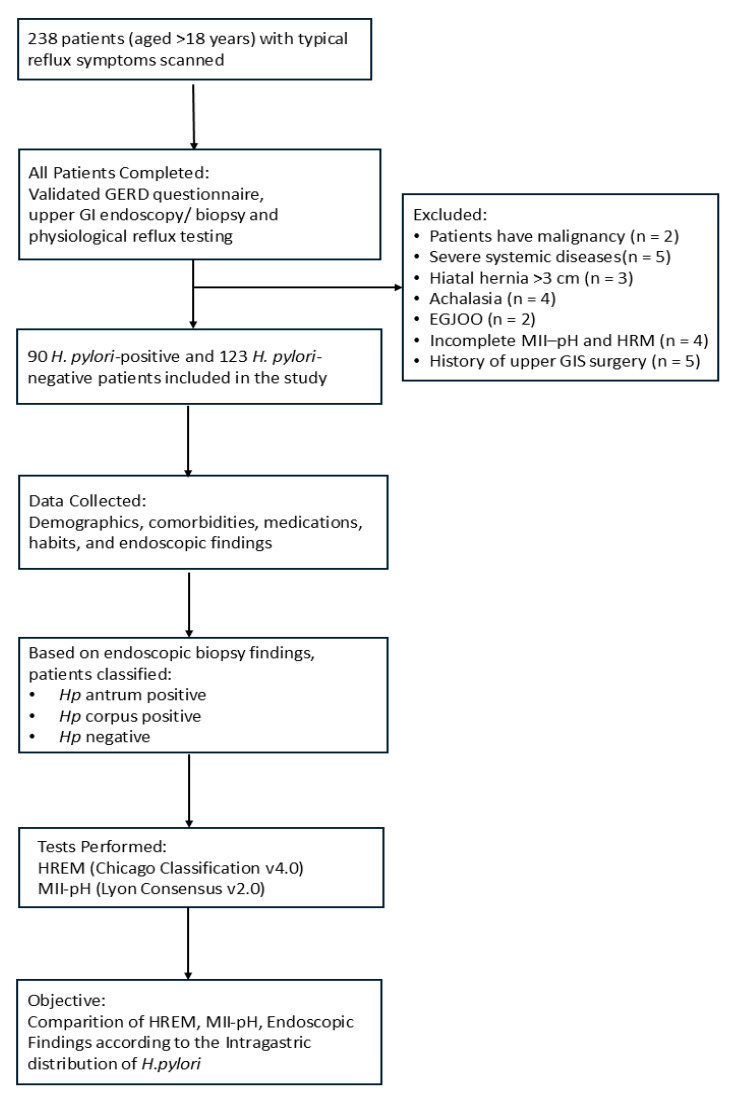
A flowchart of the study design. GERD, gastroesophageal reflux disease; GI, gastrointestinal; *H. Pylori*, *Helicobacter pylori*; HREM, high-resolution esophageal manometry; MII-pH, multichannel intraluminal impedance–pH monitoring; EGJOO, esophagogastric junction outflow obstruction.

Esophageal motility and coordination of the gastroesophageal junction were evaluated using 10 separate swallowing sequences, each consisting of 5 mL of room-temperature water. Swallows associated with physiological artifacts such as coughing, belching, or consecutive swallows, which were deemed technically unreliable, were excluded from the analysis; only 10 valid single swallows were considered. According to the Chicago Classification v4.0., IEM is diagnosed when ≥70% of swallows show ineffective peristaltic activity (low-amplitude or non-transmitted pressure waves) or when more than 50% of swallows exhibit weak contractions [18]. Additionally, disruptions in peristaltic waves were assessed as peristaltic breaks (PBs); breaks longer than 5 cm were classified as major peristaltic breaks, which are important for the qualitative analysis of esophageal coordination. The study flow chart is presented in Figure 1.

### 2.8. Statistical Analysis

All statistical analyses were performed using IBM SPSS version 29.0 (IBM Corp., Armonk, NY, USA). Data normality was assessed using the Kolmogorov–Smirnov and Shapiro–Wilk tests. Normally distributed continuous variables are expressed as mean ± standard deviation, whereas non-normally distributed variables are presented as median and interquartile range. Categorical variables are summarized as frequencies and percentages. Group comparisons were conducted using the Kruskal–Wallis test, followed by Dunn’s test for post hoc pairwise comparisons. Associations between categorical variables were evaluated using the Chi-square test with Bonferroni correction. A *p*-value of less than 0.05 was considered statistically significant.

## 3. Results

A total of 213 patients presenting with typical GERD symptoms underwent comprehensive upper endoscopy, esophageal manometry, and MII-pH. Among them, 90 were positive for *H. Pylori*, including 60 with antral colonization, three with corpus-restricted involvement, and 27 with pangastric distribution (grouped as corpus-positive). The remaining 123 patients were *H. Pylori*-negative.

### 3.1. Demographic and Clinical Characteristics

A significant sex-based difference was observed between groups: male predominance was higher in the *H. Pylori*-negative group (*p* = 0.012), while females were more prevalent in the antrum-positive group (*p* = 0.019). No significant gender difference was found in the corpus-positive group. Baseline characteristics stratified by intragastric *H. pylori* distribution are presented in Table 1.

There were no statistically significant intergroup differences in median age (*p* = 0.434), body weight (*p* = 0.851), or body mass index (*p* = 0.635).

The relationship between smoking status and *H. pylori* colonization, stratified by intragastric localization (negative, corpus-predominant, antrum-predominant), was analyzed, revealing no statistically significant differences in *H. pylori* status among current, former, and non-smokers (*p* = 0.762). However, it is noteworthy that *H. pylori*-negative individuals in our cohort demonstrated significantly longer smoking duration and higher smoking intensity (*p* = 0.018). Additionally, total tobacco exposure—measured in both daily cigarette count and pack-years—was significantly greater in *H. pylori*-negative individuals than in antrum-positive patients (*p* = 0.007 and *p* = 0.024, respectively). Alcohol consumption did not differ across groups (*p* = 0.584).

### 3.2. Symptomatology

There were no significant differences among groups in the distribution of typical or atypical GERD-related symptoms such as heartburn, regurgitation, dysphagia, chest or stomach pain, belching, cough, or hoarseness (all *p* > 0.05). A significantly higher proportion of patients with *H. pylori* positivity reported frequent nausea compared to *H. pylori*-negative subjects (48.9% vs. 29.3%, respectively; *p* = 0.004). Furthermore, the distribution of *H. pylori* colonization sites (corpus vs. antrum) was also significantly associated with the presence of frequent nausea (*p* = 0.007). Among the groups, patients with antral *H. pylori* colonization exhibited the highest rate of nausea (53.3%), followed by those with corpus involvement (40.0%) and *H. pylori*-negative individuals (29.3%). Post hoc comparisons confirmed that the frequency of nausea was significantly higher in patients with antral *H. pylori* colonization compared to *H. pylori*-negative patients (*p* = 0.005). Vomiting rates did not differ significantly between groups (*p* = 0.194).

### 3.3. Comorbidities

Comorbid conditions were present in 53 *H. pylori*-positive and 35 *H. pylori*-negative patients (*p* = 0.539). No statistically significant associations were observed between *H. pylori* status and most comorbidities, including hypertension, diabetes mellitus, asthma, depression, and hypothyroidism (*p* > 0.05 for all). However, chronic laryngitis was significantly more frequent in antrum-positive patients (20%) than in *H. pylori*-negative patients (7.3%; *p* = 0.035), and was the only comorbidity to show a significant association with *H. pylori* localization (*p* = 0.039) (Table 1). All included patients presented with clinically typical reflux symptoms, while data on chronic laryngitis—considered one of the atypical clinical manifestations of GERD—were obtained from the GERD questionnaire and patient medical history.

### 3.4. Endoscopic Findings

Endoscopic evaluation revealed no significant relationship between *H. pylori* distribution and the presence or severity of esophagitis as classified by the LA classification (*p* = 0.418). Most patients across all groups exhibited no esophagitis (82.9% in *H. pylori* -negative, 80% in corpus-positive, and 91.7% in antrum-positive patients) (Table 2).

The number of patients with hiatal hernias smaller than 3 cm was 21 (10%). To evaluate potential confounding effects, subgroup analyses were conducted comparing reflux and motility parameters according to hiatal hernia status and the intragastric distribution of *H. pylori* (negative, antrum-predominant, corpus-predominant). No statistically significant associations were found between the presence of hiatal hernia and *H. pylori* colonization in the antrum (*p* = 0.150), corpus (*p* = 1.000), or overall *H. pylori* positivity (*p* = 0.117). Similarly, no significant relationship was observed between hiatal hernia and the localization pattern of *H. pylori* (*p* = 0.123). Further analysis confirmed that the distribution of *H. pylori* subgroups did not differ significantly between patients with and without small hiatal hernias (*p* = 0.123). Moreover, no interaction effect was identified between hiatal hernia presence and H. pylori localization on key reflux parameters, including acid exposure time, DeMeester score, and esophageal motility metrics (all *p* < 0.001). These findings suggest that hiatal hernias smaller than 3 cm do not significantly confound the association between *H. pylori* intragastric distribution and reflux physiology in this cohort.

### 3.5. Twenty-Four-Hour Impedance–pH Monitoring

Comprehensive impedance–pH parameters—including SI, SAP, DeMeester score, AET, RE, and MNBI—did not significantly differ across *H. pylori* subgroups (all *p* > 0.05). These findings suggest that *H. pylori* status does not significantly impact esophageal acid burden or reflux parameters as measured by impedance–pH monitoring (Table 3). In *H. pylori*-negative patients, the median SI was 12.5 (0–50) and the median SAP was 70.0 (0–99.8), whereas in *H. pylori*-positive patients, the median SI was 0 (0–40.73) and the median SAP was 66.9 (0–97.4). There were no statistically significant differences in SI values between *H. pylori*-positive and *H. pylori*-negative patients (*p* = 0.327), nor in SAP values (*p* = 0.598). Furthermore, when stratified by *H. pylori* intragastric localization, patients with antrum-predominant and corpus-predominant infection also showed no significant differences in SI or SAP (*p* > 0.05 for both comparisons). These results suggest that *H. pylori* presence and distribution do not significantly affect the symptom–reflux correlation profile in GERD patients. The median bolus exposure time was 0.5 s (0.2–1.0) in *H. pylori*-negative patients, 0.8 s (0.3–1.6) in those with corpus-predominant *H. pylori* colonization, and 0.45 s (0.1–1.0) in patients with antral colonization. However, no statistically significant differences were observed among the groups (*p* = 0.705). These findings suggest that neither the presence nor the topographical distribution of *H. pylori* within the stomach significantly modulates esophageal bolus exposure in GERD patients. Accordingly, *H. pylori* does not appear to exert a measurable influence on either acid or non-acid reflux, nor on the physiological or perceptual correlation between reflux episodes and symptom manifestation.

### 3.6. High-Resolution Esophageal Manometry (HREM)

Analysis of HREM data (Chicago Classification v4.0) showed no significant differences in integrated relaxation pressure, distal contractile integral (DCI), distal latency, LES pressure, or peristaltic break length among groups; additionally, the distribution of normal motility versus IEM did not differ significantly (*p* = 0.931). However, contractile front velocity (CFV) was significantly reduced in antrum-positive patients compared with *H. pylori*-negative individuals (*p* = 0.002). The difference between antrum-positive and corpus-positive groups approached significance (*p* = 0.052), suggesting a potential association between antral colonization and altered esophageal contractility (Table 4). On the other hand a Mann–Whitney U test was conducted to evaluate whether CFV differed between patients with and without dysphagia. The results demonstrated no statistically significant difference in CFV values between the two groups (*p* = 0.963). These results suggest that the presence or topographical distribution of *H. pylori* within the stomach does not significantly modulate the perceptual or physiological correlation between reflux events and symptoms in GERD. Additionally, the lack of significant variation in bolus exposure time among *H. pylori*-negative patients and those with corpus- or antrum-predominant infection (*p* = 0.705) further supports the conclusion that *H. pylori* does not exert a measurable influence on esophageal acid or non-acid exposure as it relates to reflux episodes.

## 4. Discussion

The relationship between *H. pylori* infection and GERD remains controversial, with studies reporting conflicting findings—ranging from a protective effect to no association or even a worsening of reflux symptoms [4,5,6,7,12,21,22,23,24,25]. However, the vast majority of existing studies have focused almost exclusively on the presence or absence of *H. pylori* in relation to endoscopically defined erosive esophagitis. In contrast, investigations that incorporate advanced physiological testing—such as impedance–pH monitoring and HREM—to evaluate the functional impact of *H. pylori* in both erosive and non-erosive reflux disease are notably scarce. Furthermore, very few studies have examined whether the intragastric distribution of the bacterium—whether localized to the antrum, corpus, or diffusely (pangastric)—might differentially affect reflux pathophysiology. This gap in the literature limits our understanding of the nuanced role that *H. pylori* may play in GERD beyond mere presence or absence, particularly regarding its potential influence on esophageal acid exposure and motility.

In our study, a notable sex-based difference was observed: male predominance was found in the *H. pylori*-negative group, whereas females were more prevalent in the antrum-positive group. While global data suggest a modest male predominance in *H. pylori* infection, our results may reflect regional, anatomical, or demographic variations. Although many epidemiological studies have reported associations between *H. pylori* infection and sex, these associations often vary depending on geographic location, age group, and socioeconomic status [1,26,27,28,29,30]. In Turkey, available data generally show comparable seroprevalence rates between males and females, including among children [30,31]. However, consistent with international findings, some national studies also report slightly higher susceptibility in males [3,28]. These inconsistencies highlight the complexity of sex-related factors in *H. pylori* epidemiology and underscore the need for more comprehensive, standardized studies to better elucidate the role of sex in infection risk and disease progression.

In the present study, we comprehensively evaluated the associations between *H. pylori* intragastric localization and reflux characteristics based on the Lyon Consensus 2.0 [16] and the Chicago Classification v4.0 [18]. Our findings reveal that *H. pylori* was not significantly associated with acid exposure or erosive esophagitis. Although no statistically significant differences were observed in MII-pH parameters among the *H. pylori* subgroups, certain non-significant trends may hold clinical relevance. Notably, patients with corpus-predominant *H. pylori* colonization exhibited a numerically higher median SI and a slightly greater proportion of pathological DeMeester scores compared to those with antral-predominant infection. Additionally, both REs and AET appeared modestly elevated in the corpus-predominant group. While these differences did not reach statistical significance, this may be attributed, at least in part, to the relatively small sample size in some subgroups—particularly the corpus-predominant cohort. Therefore, these observations merit further investigation in larger, adequately powered studies to clarify whether *H. pylori* localization influences esophageal acid exposure and reflux symptom correlation in a clinically meaningful way. In addition, antral *H. pylori* was linked to higher rates of nausea and chronic laryngitis. Nausea is a nonspecific symptom frequently associated with gastric dysmotility, mucosal inflammation, or neurohumoral mechanisms. Chronic antral-predominant gastritis may increase gastrin-mediated acid production and delay gastric emptying, leading to persistent nausea symptoms [32,33]. Moreover, *H. pylori*-induced inflammatory changes in the enteric nervous system may contribute to dyspeptic features, especially nausea [34]. While some studies demonstrate associations between *H. pylori* and upper GI symptoms [32,34,35,36], others report contradictory findings, likely reflecting individual variability and differences in bacterial virulence or host response [13,14]. Notably, antral *H. pylori* infection was associated with an increased prevalence of chronic laryngitis in our study. Although refluxate frequency may not differ, antral colonization may enhance gastric acidity, increasing the injurious potential of REs, particularly in the laryngopharyngeal region [37]. Furthermore, the presence of *H. pylori* DNA in laryngeal tissues has raised questions about direct colonization or transitory presence via microaspiration [38,39]. Nevertheless, the causality remains debatable. While some studies refute any link [40], others support a role for *H. pylori* in laryngeal inflammation [41,42]. Although all patients presenting with typical reflux symptoms underwent objective evaluation using MII-pH monitoring—which allows for comprehensive assessment of both distal and proximal reflux—the possibility that chronic laryngitis may represent laryngopharyngeal reflux rather than classical GERD cannot be entirely excluded. This distinction may introduce diagnostic heterogeneity and should be acknowledged as a potential limitation in interpreting the association between chronic laryngitis and *H. pylori* status.

The relationship between smoking and *H. pylori* infection remains controversial, with studies reporting divergent results. While Bateson et al. [43] suggested that the immunosuppressive effects of smoking may increase susceptibility to *H. pylori* infection and the risk of peptic ulcer disease, Yu et al. [44] demonstrated that chronic and heavy smoking impairs the success of eradication therapies. In contrast, research conducted by Sirkeci et al. in Cyprus found no significant association between cigarette smoking and *H. pylori* infection, though waterpipe use was linked to higher transmission rates [45]. Several other studies have similarly reported no meaningful correlation between smoking habits and *H. pylori* status [46,47]. In our study, the relationship between smoking status and *H. pylori* colonization, categorized by intragastric localization, was analyzed, revealing no statistically significant differences across smoking groups (current, former, and non-smokers) in terms of *H. pylori* status. Interestingly, our analysis revealed significantly longer smoking duration and higher smoking intensity among *H. pylori*-negative individuals—a finding that contrasts with the majority of existing literature. This paradoxical result may indicate a potential inverse association between cumulative smoking exposure and *H. pylori* colonization. However, this relationship may be influenced by unmeasured confounding factors, such as dietary habits, socioeconomic status, and host immune response variability, which were not accounted for in the current analysis. Moreover, regional or population-specific characteristics may further modulate this association. Taken together, these findings underscore the need for prospective studies that integrate detailed lifestyle evaluations and immunological profiling to elucidate the underlying mechanisms of this complex interplay.

Several earlier studies have suggested an inverse association between *H. pylori*—particularly corpus-predominant gastritis—and erosive esophagitis, primarily based on endoscopic detection alone [4,5,6]. Others have reported increased esophagitis severity and higher prevalence of Barrett’s esophagus in *H. pylori*-negative individuals, proposing mechanisms such as acid suppression due to atrophic gastritis or alterations in gastric motility [21]. Conversely, in some populations, eradication of *H. pylori* has been linked to increased gastric acid secretion and a heightened risk of reflux symptoms [8,9]. However, a major limitation of many of these earlier studies is their reliance solely on endoscopic findings, without incorporating functional diagnostic tools such as impedance–pH monitoring or high-resolution manometry to assess reflux burden and esophageal function. In contrast, our study comprehensively evaluated reflux not only through endoscopy but also by integrating impedance–pH and manometric parameters, allowing a more physiologically robust assessment. Consistent with findings from several Turkish cohorts [7,22], we observed no significant association between *H. pylori* infection and GERD severity, either endoscopically or in terms of objective reflux metrics. Although no statistically significant differences in esophagitis severity were observed among the *H. pylori* subgroups, the small number of patients with advanced esophagitis (LA grade C/D) may have limited the statistical power to detect potential associations. Importantly, in accordance with the Lyon Consensus 2.0, the presence of LA grade C or D esophagitis constitutes definitive evidence of pathological reflux, thus precluding the need for further physiological testing such as impedance–pH monitoring. This diagnostic criterion likely accounts for the underrepresentation of LA-C and D patients in our impedance–pH analysis and should be considered when interpreting the findings.

The role of *H. pylori* virulence factors such as CagA, VacA, and CagE in modulating acid secretion and influencing GERD development has been previously proposed [13,14]. However, our retrospective design did not allow for such molecular analyses. This limitation underscores the need for future studies incorporating genotypic profiling to clarify the bacterium’s contribution to reflux pathophysiology.

Previous studies have reported inconsistent findings regarding the impact of *H. pylori* infection on pH-metry and esophageal manometry parameters. In a large-scale study by Gisbert et al., no significant association was observed between *H. pylori* infection and abnormal acid exposure, and LES pressure remained similar between infected and uninfected groups [12]. Likewise, Awad et al. reported no differences in LES pressure, peristaltic wave duration, or the presence of reflux esophagitis based on *H. pylori* status [48]. In contrast, Zhao et al. demonstrated that DCI values decreased and reflux parameters worsened following *H. pylori* eradication, suggesting a potential protective effect of the bacterium [10]. Similarly, the absence of *H. pylori* has been identified as an independent risk factor for erosive esophagitis in other cohorts [24]. Consistently, recent comprehensive meta-analyses indicate that while *H. pylori* infection may reduce GERD risk, eradication significantly increases the incidence of reflux esophagitis [49]. However, findings remain heterogeneous. For instance, Verma et al. found persistent reflux symptoms and no significant changes in manometric measures after eradication in a small prospective study [11]. The association between *H. pylori* and esophageal motility disorders such as IEM has also yielded mixed results. Zhao et al. reported an increase in IEM frequency and reductions in DCI and peristaltic activity after eradication, with fewer peristaltic breaks observed in *H. pylori*-positive individuals—possibly indicating a modulatory effect of the infection on esophageal motility [10]. Conversely, Grande et al., found no differences in LES pressure, wave amplitude, or length with respect to *H. pylori* status in a large Italian cohort [50]. Consistent with the latter, our study found no significant association between *H. pylori* infection and the prevalence of normal motility or IEM. However, a key finding emerged regarding CFV: patients with antral *H. pylori* positivity exhibited significantly lower CFV compared with *H. pylori*-negative individuals. This reduced peristaltic wave velocity may reflect altered esophageal neuromotor function, potentially mediated by chronic antral inflammation affecting vagal or enteric pathways [25]. It is hypothesized that irregular or suppressed acid secretion induced by *H. pylori* may provoke compensatory or inhibitory esophageal motor responses, thereby slowing CFV [24]. Conversely, the elevated CFV observed in *H. pylori*-negative individuals may represent an adaptive response to increased gastric acid exposure, suggesting that CFV could serve as a subtle physiological indicator of the indirect influence of *H. pylori* on esophageal motor function. In contrast, our observation of reduced CFV in patients with antral *H. pylori* colonization may reflect early or subclinical impairment in esophageal peristalsis. Although this reduction was statistically significant, it did not correspond with a higher prevalence of dysphagia. Moreover, no significant difference in CFV was found between patients with and without dysphagia, indicating that the observed variation in CFV may lack clinical relevance in terms of symptom perception. These findings suggest that the reduction in CFV may represent a subclinical motility alteration rather than overt esophageal dysmotility. Prospective studies incorporating high-resolution manometry and bolus clearance metrics are warranted to determine the prognostic or therapeutic implications of CFV changes in this subset of GERD patients.

Although statistically significant, some of the observed differences—such as reduced CFV in antrum-positive patients and the increased frequency of nausea—did not coincide with alterations in reflux burden, symptom correlation indices, or esophageal acid exposure. These findings, while potentially physiologically relevant, may not reflect clinically meaningful differences in terms of symptom severity, diagnostic classification, or treatment decisions. As such, they should be interpreted with caution and considered hypothesis-generating rather than conclusive.

Our findings do not endorse the routine eradication of *H.pylori* in GERD patients based solely on reflux burden or the presence of esophagitis. Instead, eradication strategies should be individualized, taking into account the patient’s symptom profile and endoscopic findings, particularly the presence of peptic ulcers, premalignant gastric lesions, or a family history of gastric malignancy, in line with established *H. pylori* eradication guidelines.

The heterogeneity observed in previous studies likely stems from differences in study design, sample characteristics, and the use or absence of objective diagnostic tools such as MII-pH monitoring and HREM. Our study, by incorporating both functional testing and localized *H. pylori* analysis, offers a more nuanced perspective on the complex and multifactorial interplay between *H. pylori* infection and esophageal physiology.

### 4.1. Study Limitations

This study has several limitations. First, the lack of data on *H. pylori* virulence factors and the absence of recorded PPI use during endoscopic evaluations may have confounded our findings. PPIs significantly influence acid secretion, and their uncontrolled use could introduce bias. Additionally, the retrospective design limits causal inference and may have introduced selection bias. Second, an important methodological limitation of this study is the use of a water-perfused manometry system rather than a solid-state high-resolution manometry catheter. While water-perfused systems are clinically acceptable and widely used, they may underestimate subtle esophageal motility abnormalities due to their lower temporal resolution and sensitivity. This may limit the detection of minor differences in peristaltic function among study groups.

In this study, typical reflux symptoms were assessed using the validated GERD-Q questionnaire [15]. However, a key limitation lies in the absence of a dedicated instrument specifically designed to evaluate dyspeptic symptoms. This constraint may have impeded our ability to accurately distinguish between nausea attributable to GERD and that arising from functional dyspepsia (FD). Although recent meta-analyses indicate that approximately 41% of patients with GERD also report symptoms consistent with FD, and nearly one-third of FD patients experience reflux-like complaints—highlighting the complex interplay and diagnostic overlap between these disorders [51]—the lack of a dyspepsia-specific tool remains a methodological shortcoming. Furthermore, while our findings suggest a potential association between antral *H. pylori* colonization and symptoms such as nausea and chronic laryngitis, these observations should be interpreted with caution. The retrospective nature of the study, along with potential confounding factors—including functional dyspepsia, smoking status, and chronic rhinitis, which were assessed but may still influence symptom presentation—limits the ability to infer causality. Finally, it is important to acknowledge that this study was conducted in Turkey, a country with a markedly high prevalence of *H. pylori* infection. As such, the generalizability of our findings to populations with a lower prevalence may be limited. Regional variations in *H. pylori* strain virulence (e.g., CagA, VacA positivity), host genetic susceptibility, environmental exposures, and healthcare practices may all influence the interaction between *H. pylori* and GERD pathophysiology. Therefore, caution is warranted when extrapolating these results to different epidemiological settings, and further studies from diverse geographic regions are needed to validate our findings.

### 4.2. Study Strengths

Despite these limitations, to the best of our knowledge, this is one of the first studies to comprehensively assess the impact of intragastric *H. pylori* distribution on esophageal manometry and 24 h pH–impedance monitoring parameters. The inclusion of both erosive and non-erosive reflux disease patients, combined with objective physiological assessments, strengthens the robustness and clinical relevance of our findings. By examining the specific localization of *H. pylori* within the stomach (antrum, corpus, or pangastric), and integrating endoscopic, symptomatic, and functional data, our study offers a more nuanced perspective on the bacterium’s role in reflux pathophysiology.

### 4.3. Future Directions

To advance our understanding of the pathophysiological interactions between *H. pylori* and GERD, future research should prioritize prospective, controlled studies with larger cohorts and comprehensive clinical characterization.

The observation of longer duration and greater intensity of smoking exposure in *H. pylori*-negative patients may represent a chance finding rather than a biologically plausible association. This result should be considered exploratory, and further research is needed to determine whether smoking status influences *H. pylori* colonization or reflects underlying confounding factors.

Key methodological improvements should include systematic documentation of PPI usage, as PPI therapy can profoundly alter the gastric environment and reflux profiles. Molecular profiling of *H. pylori* strains—particularly regarding virulence factors such as CagA, VacA, and CagE—may help clarify inter-individual variability in reflux-related outcomes; furthermore, the correlation between *H. pylori* density, gastric topography, and reflux severity should be examined in more detail, potentially revealing dose-dependent or regional effects on acid secretion and esophageal function. The observed reduction in CFV among antrum-positive patients is an intriguing finding; however, its clinical significance remains uncertain. Given the lack of direct correlation with dysphagia or other symptom severity in our cohort, this result should be considered hypothesis-generating and interpreted cautiously until validated in prospective studies Assessing patients before and after eradication therapy—especially in cases of antral-predominant infection—are essential for elucidating the dynamic impact of *H. pylori* on esophageal motility parameters such as CFV and the prevalence of IEM. These investigations could offer critical insights into whether motility changes are reversible or persist beyond eradication, with potential implications for symptom management in GERD.

## 5. Conclusions

Antral *H. pylori* colonization was associated with reduced CFV, a novel observation that warrants further investigation in prospective cohorts to determine its clinical relevance. Overall, *H. pylori* status and intragastric distribution did not significantly influence esophageal acid exposure or reflux–symptom correlation in GERD patients. While these findings suggest a limited role of *H. pylori* in GERD pathophysiology, they should not be interpreted as a rationale to withhold eradication therapy, given its well-established benefits in preventing peptic ulcer disease and gastric malignancies.

## Figures and Tables

**Table 1:** Baseline demographic characteristics of patients stratified by intragastric *Helicobacter pylori* localization.

	Hp (−) (*n* = 123)	Hp Antrum (+) (*n* = 60)	Hp Corpus (+) (*n =* 30)	*p*-Value	sig. Diff.
Age	42 (36–56)	44 (34–51)	46 (39–52)	0.434	—
Weight	72.92 ± 12.75	72.5 ± 12.84	75.73 ± 16.37	0.851	—
BMI	25.65 ± 3.86	26.36 ± 4.94	26.68 ± 4.14	0.635	—
Female	51 (49) ^a^	37 (35.6) ^b^	16 (15.4) ^c^	0.034	^b^ vs. ^c^: *p* = 0.019
Male	72 (66.1) ^a^	23 (21.1) ^b^	14 (12.8) ^c^	0.034	^a^ vs. ^b^: *p* = 0.012

Statistical comparisons: Pearson’s Chi-square test was used for categorical variables; the Kruskal–Wallis test was applied for non-normally distributed continuous variables; and one-way ANOVA was used for normally distributed continuous variables. Data presentation: Continuous variables are expressed as mean ± standard deviation for normally distributed data (e.g., weight, BMI) or as median (interquartile range) for non-normally distributed data (e.g., age), as appropriate. Categorical variables (e.g., sex) are presented as number (percentage). Post hoc comparisons: Superscripts (^a^, ^b^, ^c^) indicate statistically significant differences between groups in pairwise post hoc comparisons (*p* < 0.05). Groups sharing the same superscript are not significantly different. Post hoc tests were performed using Bonferroni correction for Chi-square tests and Dunn’s test for Kruskal–Wallis tests. Abbreviations: Hp, *Helicobacter pylori*; (−), negative; (+), positive; sig. diff., significant differences. Units: Age (years); Weight (kg); BMI (kg/m^2^).

**Table 2:** Comparison of endoscopic esophagitis findings according to the intragastric distribution of *Helicobacter pylori* based on the Los Angeles Classification.

Endoscopic Appearance	Hp (−) (*n* = 123)	Hp Antrum (+) (*n* = 60)	Hp Corpus (+) (*n* = 30)	*p*-Value
No Esophagitis	102 (82.9)	55 (91.7)	24 (80.0)	0.418
LA-A Esophagitis	13 (10.6)	4 (6.7)	3 (10.0)	
LA-B, C, or D Esophagitis	8 (6.5)	1 (1.7)	3 (10.0)	

Statistical analysis: *p*-value was calculated using Pearson’s Chi-square test. Data presentation: Categorical variables are expressed as number (percentage). Findings: No statistically significant difference was observed between the intragastric distribution of *Helicobacter pylori* and the presence or severity of esophagitis. Note: The low frequency of LA–C and LA–D esophagitis cases may have limited the statistical power for subgroup analysis. Abbreviations: LA, Los Angeles classification of esophagitis; Hp, *Helicobacter pylori*; (−), negative; (+), positive.

**Table 3:** Comparison of 24 h ambulatory impedance–pH monitoring parameters based on the intragastric distribution of *Helicobacter pylori*.

Parameter	Hp (−) (*n* = 123)	Hp Antrum (+) (*n* = 60)	Hp Corpus (+) (*n* = 30)	*p*-Value
DeMeester	11.7 (3.5–26.3)	9.2 (3.5–24.4)	10.3 (3.8–27.3)	0.919
DeMeester ≥ 14.72	56 (46)	23 (38)	13 (43)	0.650
AET	3.4 (0.7–6.3)	2.3 (0.6–6.3)	2.9 (0.8–7.2)	0.697
AET < 4	67 (55)	38(63)	17 (56)	0.401
AET 4–6	21 (17)	6 (10)	2 (7)	
AET > 6	35 (28)	16 (27)	11 (37)	
RE	23 (10.0–40.0)	28.5 (12.5–45.8)	28 (18.2–49.5)	0.523
RE < 40	93 (75)	43 (72)	20 (67)	0.627
RE 40–80	22 (18)	14 (23)	9 (30)	
RE > 80	8 (7)	3 (5)	1 (3)	
SI	12.5 (0.0–99.8)	2.5 (0.0–42.0)	70.0 (0.0–99.0)	0.500
SAP	70.0 (0.0–99.0)	70.3 (0.0–98.0)	59.7 (0.0–99.0)	0.693
MNBI (Ω)	2950 (1950–4200)	3415 (2265–4585)	2825 (1850–3697)	0.213
MNBI < 1500	13 (11)	6 (10)	4 (13)	0.732
MNBI 1500–2500	39 (32)	14 (23)	7 (23)	
MNBI > 2500	71 (57)	40 (67)	19 (64)	

Statistical tests: Continuous variables were compared using the Kruskal–Wallis test, while categorical variables were analyzed using Pearson’s Chi-square test. A *p*-value of <0.05 was considered statistically significant. Data presentation: Continuous variables (SI, SAP, DeMeester score, number of reflux episodes, MNBI) are presented as median (interquartile range). Categorical variables (AET < 4, AET 4–6, AET > 6; RE < 40, RE 40–80, RE > 80; MNBI < 1500, MNBI 1500–2500, MNBI > 2500) are presented as number (percentage). Abbreviations: AET, acid exposure time; DeMeester, DeMeester score; MNBI, mean nocturnal baseline impedance; RE, number of reflux episodes; SAP, symptom association probability; SI, symptom index; Hp, *Helicobacter pylori*; (−), negative; (+), positive.

**Table 4:** Comparison of esophageal high-resolution manometry parameters according to the intragastric distribution of *Helicobacter pylori*.

Parameter	Hp (−) (*n* = 123)	Hp Antrum (+) (*n =* 60)	Hp Corpus (+) (*n =* 30)	*p*-Value	Sig. Diff.
IRP	7.0 (4.0–11.0)	9.0 (5.0–12.0)	6.5 (3.0–12.0)	0.386	—
DCI	708.0 (360.0–1179.0)	640.0 (342.0–886.0)	599.0 (395.0–926.0)	0.603	—
CFV	4.6 (3.6–5.8) ^a^	4.0 (3.0–4.5) ^b^	4.4 (3.8–5.5) ^c^	**0.002**	**^a^ vs. ^b^: *p* = 0.002** ^b^ vs. ^c^: *p* = 0.052 ^a^ vs. ^c^: *p* = 1.000
PB	2.0 (0.6–4.0)	1.7 (0.5–3.6)	1.9 (0.7–4.7)	0.584	—
DL	7.0 ± 1.00	7.2 ± 0.82	7.0 ± 1.22	0.353	—
LES *p*.	18.4 ± 10.3	20.0 ± 7.8	20.0 ± 9.9	0.839	—
Normal Motility	82 (67)	41 (70)	21 (70)	0.931	—
IEM	40 (33)	18 (30)	9 (30)		

Statistical tests: Continuous variables were compared using the Kruskal–Wallis test for non-normally distributed data (presented as median [interquartile range, IQR]) and one-way ANOVA for normally distributed data (mean ± standard deviation, SD). Categorical variables were analyzed using Pearson’s Chi-square test. Data presentation: IRP, DCI, CFV, and PB are shown as median (IQR); DL and LES pressure are presented as mean ± SD; categorical variables (normal motility and IEM) are shown as number (percentage). Post hoc analysis: Superscripts (^a^, ^b^, ^c^) indicate statistically significant differences between groups (*p* < 0.05). Groups sharing the same superscript are not significantly different. Post hoc pairwise comparisons for CFV were conducted with Bonferroni correction. Abbreviations: IRP, integrated relaxation pressure; DCI, distal contractile integral; CFV, contractile front velocity; PB, peristaltic break; DL, distal latency; LES *p*., lower esophageal sphincter pressure; IEM, ineffective esophageal motility; Hp, *Helicobacter pylori*; (−), negative; (+), positive.

## Data Availability

The data presented in this study are available on request from the corresponding author.

## Abbreviations

The following abbreviations are used in this manuscript.

AET	Acid exposure time
CFV	Contractile front velocity
DCI	Distal contractile integral
FD	Functional dyspepsia
GERD	Gastroesophageal reflux disease
GI	Gastrointestinal
*H. pylori*	*Helicobacter pylori*
HREM	High-resolution esophageal manometry
IEM	Ineffective esophageal motility
LA	Los Angeles classification of esophagitis
LES	Lower esophageal sphincter
MII-pH	Multichannel intraluminal impedance–pH monitoring
MNBI	Mean nocturnal baseline impedance
PPI	Proton pump inhibitor
RE	Number of reflux episodes
